# The Selective Class IIa Histone Deacetylase Inhibitor TMP195 Resensitizes ABCB1- and ABCG2-Overexpressing Multidrug-Resistant Cancer Cells to Cytotoxic Anticancer Drugs

**DOI:** 10.3390/ijms21010238

**Published:** 2019-12-29

**Authors:** Chung-Pu Wu, Sabrina Lusvarghi, Jyun-Cheng Wang, Sung-Han Hsiao, Yang-Hui Huang, Tai-Ho Hung, Suresh V. Ambudkar

**Affiliations:** 1 Graduate Institute of Biomedical Sciences, College of Medicine, Chang Gung University, Tao-Yuan 330, Taiwan; showtime983@gmail.com (J.-C.W.); johnson170_ya@hotmail.com (S.-H.H.); yanghui.huang01@gmail.com (Y.-H.H.); 2 Department of Physiology and Pharmacology, College of Medicine, Chang Gung University, Tao-Yuan 330, Taiwan; 3 Department of Obstetrics and Gynecology, Taipei Chang Gung Memorial Hospital, Taipei 10507, Taiwan; thh20@adm.cgmh.org.tw; 4 Laboratory of Cell Biology, Center for Cancer Research, National Cancer Institute, NIH, Bethesda, MD 20892, USA; sabrina.lusvarghi@nih.gov (S.L.); ambudkar@mail.nih.gov (S.V.A.); 5 Department of Chinese Medicine, College of Medicine, Chang Gung University, Tao-Yuan 333, Taiwan

**Keywords:** TMP195, histone deacetylase inhibitor, chemoresistance, modulators, P-glycoprotein, breast cancer resistance protein

## Abstract

Multidrug resistance caused by the overexpression of the ATP-binding cassette (ABC) proteins in cancer cells remains one of the most difficult challenges faced by drug developers and clinical scientists. The emergence of multidrug-resistant cancers has driven efforts from researchers to develop innovative strategies to improve therapeutic outcomes. Based on the drug repurposing approach, we discovered an additional action of TMP195, a potent and selective inhibitor of class IIa histone deacetylase. We reveal that in vitro TMP195 treatment significantly enhances drug-induced apoptosis and sensitizes multidrug-resistant cancer cells overexpressing ABCB1 or ABCG2 to anticancer drugs. We demonstrate that TMP195 inhibits the drug transport function, but not the protein expression of ABCB1 and ABCG2. The interaction between TMP195 with these transporters was supported by the TMP195-stimulated ATPase activity of ABCB1 and ABCG2, and by in silico docking analysis of TMP195 binding to the substrate-binding pocket of these transporters. Furthermore, we did not find clear evidence of TMP195 resistance conferred by ABCB1 or ABCG2, suggesting that these transporters are unlikely to play a significant role in the development of resistance to TMP195 in cancer patients.

## 1. Introduction

The overexpression of one or more multidrug resistance (MDR)-linked ATP-binding cassette (ABC) drug transporters, such as ABCB1 (P-glycoprotein, MDR1), ABCC1 (multidrug-resistance protein 1, MRP1), and ABCG2 (breast cancer resistance protein (BCRP), MXR), continues to pose a significant challenge to cancer chemotherapy [1,2,3,4]. These transporters utilize ATP hydrolysis energy to actively efflux a large variety of chemotherapeutic drugs out of cancer cells and away from their intracellular drug targets. As a result, these cancer cells become insensitive to a broad spectrum of anticancer drugs, including conventional chemotherapeutic agents and protein kinase inhibitors [5,6], and consequently lead to treatment failure in solid tumors and blood cancers [4,7]. Therefore, resolving therapeutic difficulties associated with the overexpression of ABC drug transporters has great clinical significance.

Despite the many innovative strategies that have been proposed [8,9,10], blocking the drug efflux system with a competitive inhibitor is still considered by many to be the most direct and practical approach to reverse MDR mediated by ABC drug transporters [11,12,13]. Regrettably, due to problems associated with toxicity and adverse drug–drug interactions of synthetic inhibitors, there is currently no US Food and Drug Administration (FDA)-approved modulators that can be used against multidrug-resistant cancers. Alternatively, we and others have adopted the drug repurposing (also referred to as drug repositioning) approach to discover small molecule therapeutic agents with known toxicological and pharmacological profiles, that can interact strongly with one or more transporters and resensitize multidrug-resistant cancer cells [14,15,16,17,18,19,20]. TMP195 is one of the most potent and highly selective class IIa histone deacetylase (HDAC) inhibitors identified by GlaxoSmithKline in an initiative to discover cell-active class IIa HDAC inhibitors [21]. Recently, an important study by Guerriero et al. reported that TMP195 can suppress breast cancer growth and metastasis by genomic reprogramming of monocytes and macrophages into tumoricidal cells [22]. To this end, we explored the potential mechanism and chemosensitizing effect of TMP195 in multidrug-resistant cancer cells overexpressing ABCB1, ABCC1 or ABCG2.

In the present study, we revealed an additional action of TMP195 on resensitizing ABCB1- and ABCG2-overexpressing multidrug-resistant cancer cells to multiple therapeutic drug substrates of ABCB1 and ABCG2. We then demonstrated that TMP195 enhances drug-induced apoptosis of multidrug-resistant cancer cells by modulating the drug transport function of ABCB1 and ABCG2, without affecting the protein expression of either transporter. By performing ATPase assays and drug docking analysis, we also identified the potential sites of interaction between TMP195 and the substrate-binding pockets of ABCB1 and ABCG2.

## 2. Results

### 2.1. TMP195 Reverses Multidrug Resistance Mediated by ABCB1 and ABCG2

**Figure 1:**
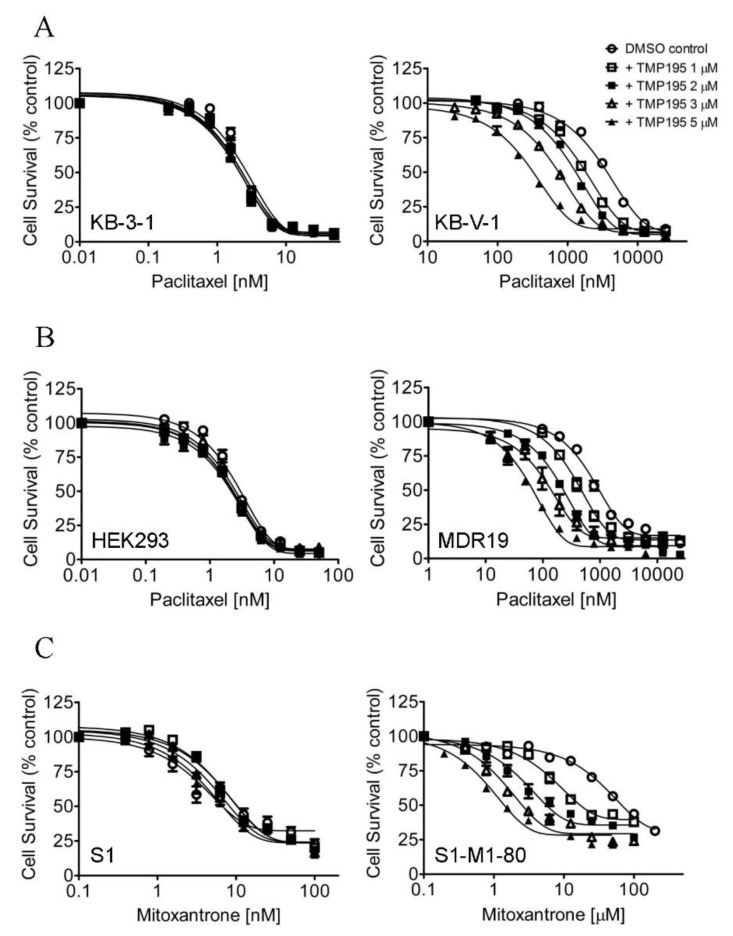

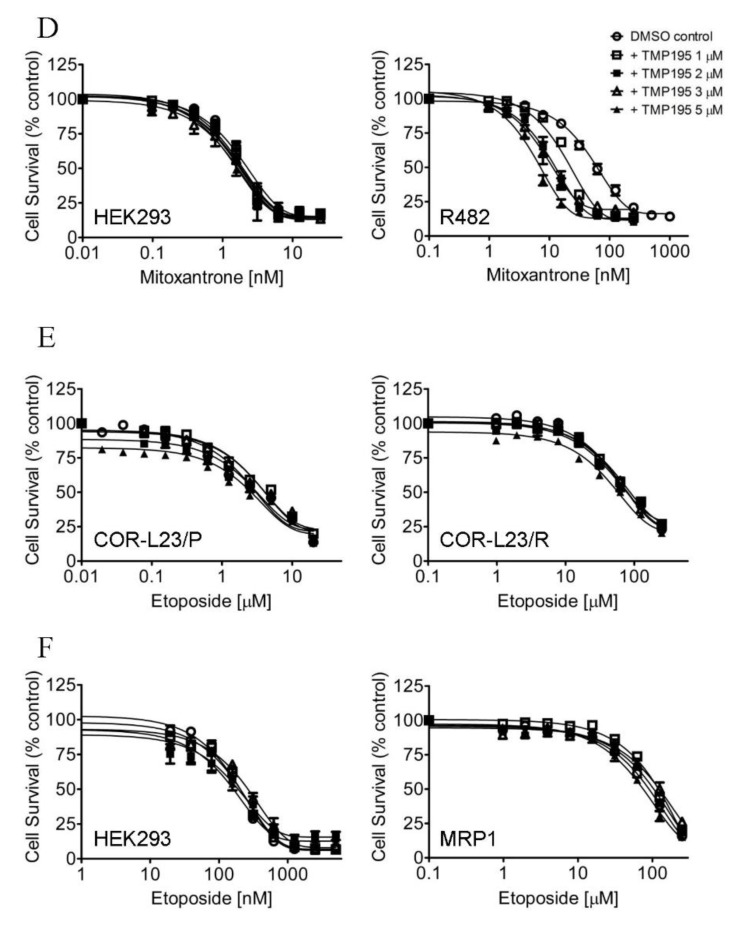
TMP195 re-sensitizes ABCB1-overexpressing cells to paclitaxel and ABCG2-overexpressing cells to mitoxantrone. The chemosensitization effect of TMP195 was examined by treating cells with increasing concentrations of paclitaxel (a known substrate drug of ABCB1), or mitoxantrone (a known substrate drug of ABCG2) or etoposide (a known substrate drug of ABCC1) in the presence of DMSO (open circles) or TMP195 at 1 μM (open squares), 2 μM (filled squares), 3 μM (open triangles) or 5 μM (filled triangles). (**A**) Drug-sensitive parental KB-3-1 (left panel) and the ABCB1-overexpressing multidrug resistance (MDR) variant KB-V-1 (right panel) human epidermal cancer cells, as well as (**B**) HEK293 cells (left panel) and HEK293 cells transfected with human ABCB1 (MDR1, right panel) were used to determine the effect of TMP195 on ABCB1-mediated paclitaxel resistance. (**C**) Drug-sensitive parental S1 (left panel) and the ABCG2-overexpressing MDR variant S1-M1-80 (right panel) human colon cancer cells, as well as (**D**) HEK293 cells (left panel) and HEK293 cells transfected with human ABCG2 (R482, right panel) were used to determine the effect of TMP195 on ABCG2-mediated mitoxantrone resistance. (**E**) Drug-sensitive parental COR-L23/P (left panel) and the ABCC1-overexpressing MDR variant COR-L23/R (right panel) human lung cancer cells, as well as (**F**) HEK293 cells (left panel) and HEK293 cells transfected with human ABCC1 (MRP1, right panel) were used to determine the effect of TMP195 on ABCC1-mediated etoposide resistance. Points, mean values from at least three independent experiments; error bars, SEM.

In order to determine the potential chemosensitization effect of TMP195, we examined the cytotoxicity of the drug substrate of ABCB1, ABCG2 or ABCC1 in the absence (open circles) or presence of TMP195 at 1 μM (open squares), 2 μM (filled squares), 3 μM (open triangles) or 5 μM (filled triangles) in cells overexpressing ABCB1, ABCG2 or ABCC1, respectively (Figure 1). First, the cytotoxicity of paclitaxel, a known drug substrate of ABCB1 [23], was determined in the drug-sensitive parental KB-3-1 human epidermal cancer cell line and its ABCB1-overexpressing MDR variant KB-V-1 (Figure 1A), as well as in HEK293 cells and HEK293 cells transfected with human ABCB1 (MDR19, Figure 1B and Table 1). As shown in Figure 1A,B, TMP195 resensitized ABCB1-overexpressing multidrug-resistant cells to paclitaxel in a concentration-dependent manner, without affecting the cytotoxicity of paclitaxel in drug-sensitive parental cells. We discovered that in addition to paclitaxel, TMP195 resensitized KB-V-1 cells and NCI-ADR-RES, an ABCB1-overexpressing MDR variant of OVCAR-8 human ovarian cancer cells, to ABCB1 drug substrates colchicine and vincristine [23,24] as well (Table 2). It is worth mentioning that verapamil, a known inhibitor (and also a transport substrate) of ABCB1, was more effective in reversing ABCB1-mediated resistance to paclitaxel and vincristine in KB-V-1 and NCI-ADR-RES cancer cells. Moreover, both verapamil and TMP195 were unable to restore the chemosensitivity of KB-V-1 and NCI-ADR-RES cancer cells to the same extent as the respective parental cells, KB-3-1 and OVCAR-8. Furthermore, TMP195 also reversed ABCG2-mediated resistance to mitoxantrone, a known drug substrate of ABCG2 [25], in S1-M1-80, an ABCG2-overexpressing MDR variant of S1 human colon cancer cells (Figure 1C), and in HEK293 cells transfected with human ABCG2 (R482, Figure 1D and Table 1). Similarly, TMP195 resensitized S1-M1-80 cells and H460-MX20, an ABCG2-overexpressing MDR variant of H460 human lung cancer cells, to SN-38 and topotecan in a concentration-dependent manner (Table 3). We found that TMP195 reversed ABCG2-mediated resistance to mitoxantrone and topotecan to a comparable level as that of Ko143, an ABCG2 benchmark inhibitor, in S1-M1-80 and H460-MX20 cancer cells. In comparison, TMP195 was least effective in reversing ABCG2-mediated resistance to SN-38 in both ABCG2-overexpressing cancer cell lines.

In contrast, TMP195 had no significant effect on ABCC1-mediated resistance to etoposide, a known drug substrate of ABCC1, in either COR-L23/R, an ABCC1-overexpressing MDR variant of COR-L23/P human lung cancer cells (Figure 1E) or in HEK293 cells transfected with human ABCC1 (MRP1, Figure 1F and Table 1). The extent of chemosensitization by TMP195, presented as the fold-reversal (FR) value [26], was calculated as the ratio of the IC_50_ value of the drug substrate alone to the IC_50_ value of the drug substrate in the presence of TMP195 (Table 1, Table 2 and Table 3). Verapamil (5 μM), Ko143 (3 μM) and MK-571 (25 μM) were used as reference inhibitors for ABCB1, ABCG2, and ABCC1, respectively. It is worth noting that verapamil induced significant cytotoxicity in cells treated with vincristine (Table 2), which is independent of ABCB1 activity. This result is consistent with previous reports of verapamil at non-toxic concentrations enhancing the cytotoxicity of vincristine in drug-sensitive cancer cells [27,28]. Our results here revealed that multidrug-resistant cancer cells overexpressing ABCB1 or ABCG2 can be significantly resensitized by TMP195.

### 2.2. TMP195 Sensitizes Cancer Cells Overexpressing ABCB1 or ABCG2 to Drug-Induced Apoptosis

**Figure 2:**
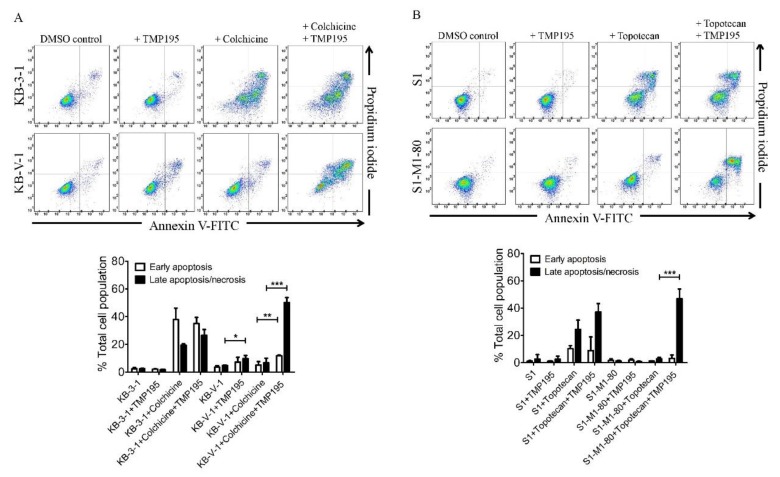
TMP195 enhances drug-induced apoptosis in ABCB1-overexpressing cancer cells and ABCG2-overexpressing cancer cells. Dot plots (upper panel) and quantification (lower panel) of (**A**) drug-sensitive KB-3-1 cells and the MDR variant KB-V-1 cells treated with either DMSO (control), 10 μM of TMP195 (+TMP195), 500 nM of colchicine (+colchicine), or a combination of 500 nM of colchicine and 10 μM of TMP195 (+colchicine +TMP195), and (**B**) drug-sensitive S1 and the MDR variant S1-M1-80 cells treated with either DMSO (control), 10 μM of TMP195 (+TMP195), 5 μM of topotecan (+topotecan) or a combination of 5 μM of topotecan and 10 μM of TMP195 (+topotecan +TMP195). Cells were treated with respective regimens, isolated, and analyzed by flow cytometry as described previously [30]. Representative dot plots and quantifications of apoptotic cell populations are presented as mean ± SD calculated from at least three independent experiments. ** *p* < 0.05; ** *p* < 0.01; *** *p* < 0.001, versus the same treatment in the absence of TMP195.

Next, we examined the effect of TMP195 on apoptosis induced by ABCB1 substrate drug colchicine and by ABCG2 substrate drug topotecan, known inducers of apoptosis [24,29], in ABCB1- and ABCG2-overexpressing human cancer cell lines. KB-3-1 and KB-V-1 cancer cells were treated with DMSO, 10 μM of TMP195, 500 nM of colchicine, or a combination of 500 nM of colchicine and 10 μM of TMP195 (Figure 2A), whereas S1 and S1-M1-80 cancer cells were treated with DMSO, 10 μM of TMP195, 5 μM of topotecan, or a combination of 5 μM of topotecan and 10 μM of TMP195 (Figure 2B) and processed as detailed in Section 4. As expected, colchicine significantly elevated the level of apoptosis in KB-3-1 cancer cells, from approximately 5% basal level to 57% of early and late apoptosis. In contrast, the effect of colchicine on ABCB1-overexpressing KB-V-1 cancer cells was significantly reduced (from approximately 8% basal level to 12% of early and late apoptosis), presumably due to ABCB1-mediated efflux of colchicine (Figure 2A). Without affecting KB-3-1 cells, TMP195 significantly increased colchicine-induced apoptosis in KB-V-1 cells, from 8% basal level to 63% of total apoptosis. Similarly, while topotecan induced substantial apoptosis of S1 cancer cells, from 4% basal level to approximately 35% of total apoptosis, topotecan had minimal effect on ABCG2-overexpressing S1-M1-80 cancer cells, likely a result of ABCG2-mediated efflux of topotecan (Figure 2B). The extent of apoptosis induced by topotecan was significantly enhanced by TMP195 in S1-M1-80 cells, from 4% basal level to 50% of early and late apoptosis. Of note, 10 μM TMP195 alone had no significant apoptotic effect in all tested cell lines, raising the possibility that TMP195 enhances drug-induced apoptosis and reverses drug resistance in cancer cells overexpressing ABCB1 or ABCG2 through modulation of the function and/or protein expression of ABCB1 and ABCG2.

### 2.3. TMP195 Attenuates the Drug Transport Function of ABCB1 and ABCG2

**Figure 3:**
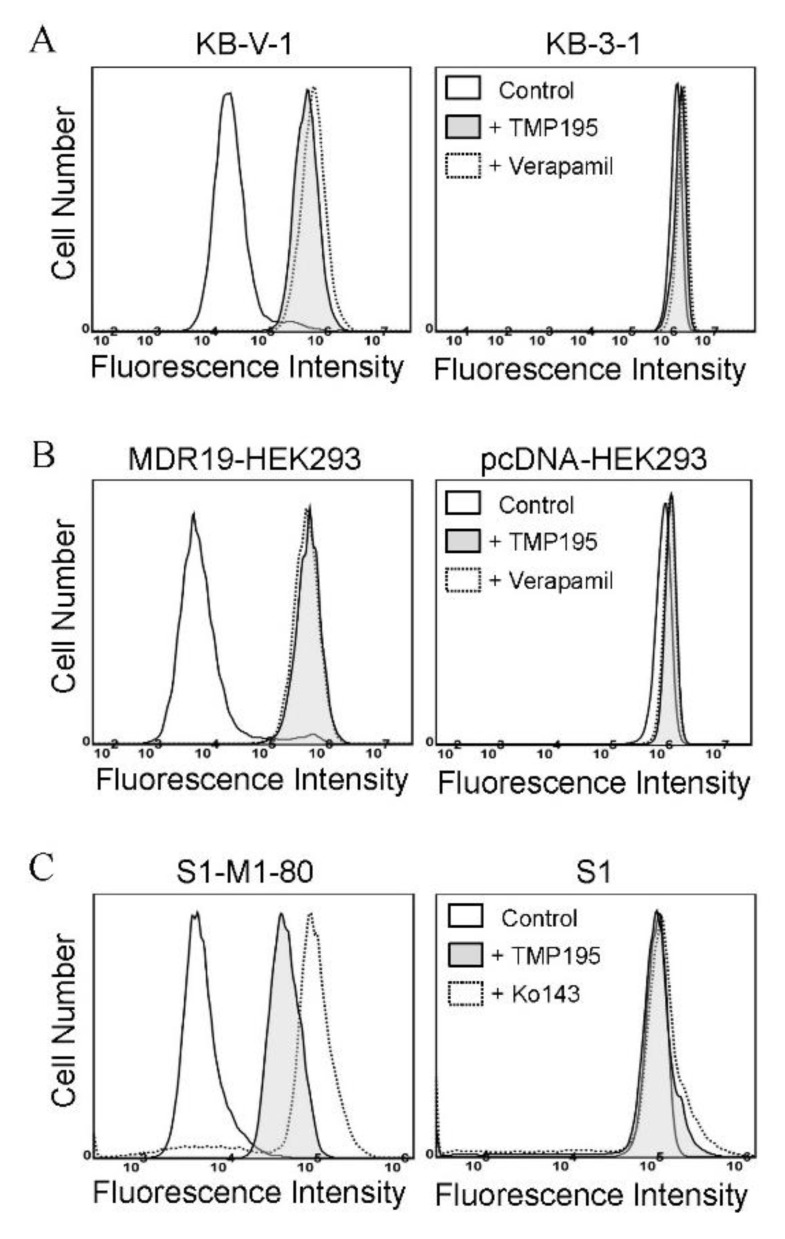

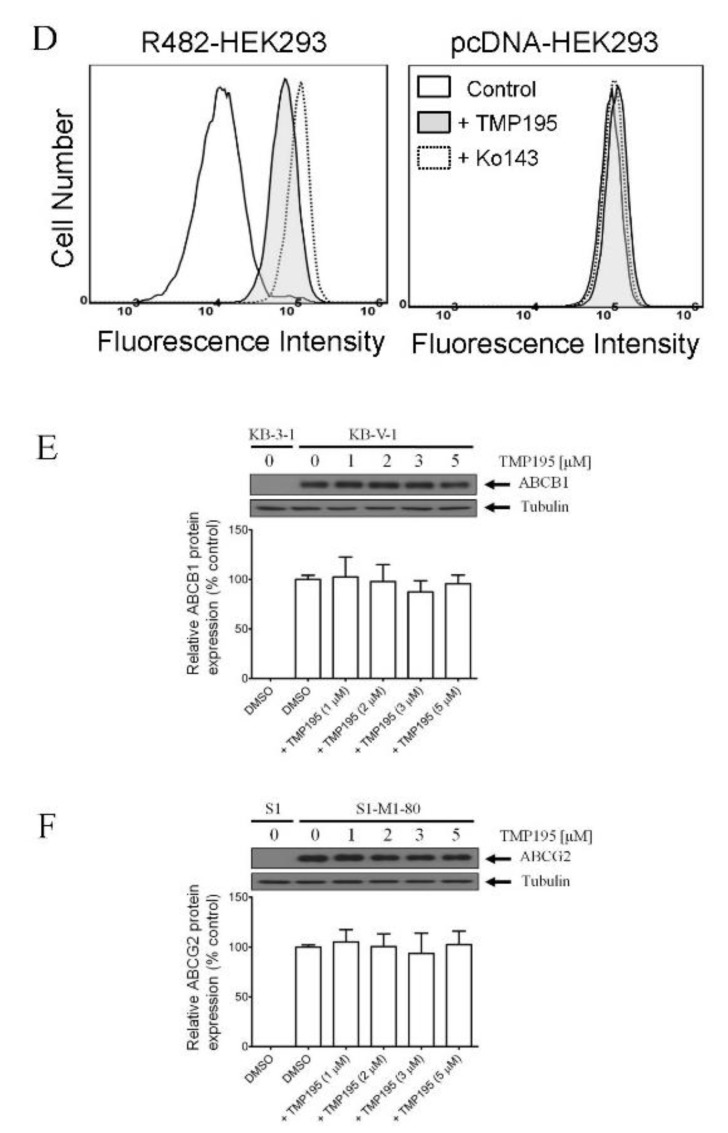
TMP195 modulates the drug efflux function, but not the protein expression, of ABCB1 and ABCG2 in human multidrug-resistant cancer cells. The effect of TMP195 on ABCB1-mediated transport of calcein-AM, a known substrate of ABCB1, was determined in (**A**) ABCB1-overexpressing KB-V-1 human epidermal cancer cells and (**B**) human HEK293 cells transfected with human ABCB1 (MDR19-HEK293), whereas the effect of TMP195 on ABCG2-mediated transport of pheophorbide A (PhA), a known substrate of ABCG2, was determined in (**C**) ABCG2-overexpressing S1-M1-80 human colon cancer cells and (**D**) HEK293 cells transfected with human ABCG2 (R482-HEK293). Respective drug-sensitive parental cell lines were used as controls (A–D, right panels). Intracellular fluorescence of calcein (A and B) or PhA (C and D) was measured in cells treated with DMSO (A–D, solid lines), 20 μM of TMP195 (A–D, filled solid lines), 20 μM of verapamil as a positive control for ABCB1 inhibition (A and B, dotted lines), or 1 μM of Ko143 as a positive control for ABCG2 inhibition (C and D, dotted lines) as indicated and analyzed by flow cytometry. Representative histograms of at least three independent experiments are shown. The effect of TMP195 on the protein expression of ABCB1 or ABCG2 was determined by treating (**E**) KB-V-1 cancer cells or (**F**) S1-M1-80 cancer cells with increasing concentrations of TMP195 (0–5 μM) as indicated for 72 h before processing for immunoblotting. α-Tubulin was used as an internal loading control. Immunoblot detection (upper panels) and quantification values (lower panels) are presented as mean ± SD calculated from at least three independent experiments.

To determine the effect of TMP195 on the drug efflux function of ABCB1 and ABCG2, we first examined the accumulation of calcein, a fluorescent product of a known ABCB1 substrate drug calcein-AM [31], in KB-V-1 and MDR19-HEK293 cells (Figure 3A,B), as well as pheophorbide A (PhA), a known fluorescent substrate of ABCG2 [32], in S1-M1-80 and R482-HEK293 cells (Figure 3C,D) and in their respective drug-sensitive parental cells. The intracellular accumulation of fluorescent substrate drugs was examined in the presence of DMSO (solid lines), 20 μM of TMP195 (shaded, solid lines) or 20 μM of verapamil as a reference inhibitor of ABCB1 (Figure 3A,B, dotted lines), and 1 μM of Ko143 as a reference inhibitor of ABCG2 (Figure 3C,D, dotted lines), and then processed as described in Section 4. We found that the intracellular accumulation of calcein in KB-V-1 (Figure 3A, left panel) and MDR19-HEK293 (Figure 3B, left panel) cells, as well as the intracellular accumulation of PhA in S1-M1-80 (Figure 3C, left panel) and R482-HEK293 (Figure 3D, left panel) cells, was greatly increased by TMP195. In contrast, TMP195 had no significant effect on the accumulation of calcein or PhA in the drug-sensitive parental cell lines (Figure 3A−D, right panels). Notably, several studies demonstrated that drug-induced transient downregulation of ABCB1 or ABCG2 is another common mechanism in which the multidrug-resistant cancer cells can become resensitized to chemotherapeutic drugs [19,33,34]. To this end, we examined the protein expression of ABCB1 in KB-V-1 cancer cells (Figure 3E) and ABCG2 in S1-M1-80 cancer cells (Figure 3F) after treating cells with increasing concentrations of TMP195 (0–5 μM) for 72 h, followed by immunoblotting as described in Section 4. We did not observe any significant effect on the protein expression of ABCB1 or ABCG2 by TMP195 in these multidrug-resistant cell lines. Our results indicate that TMP195 attenuates the drug efflux function, but not the protein expression of ABCB1 and ABCG2 in cancer cells.

### 2.4. TMP195 Stimulates the ATPase Activity of ABCB1 and ABCG2

**Figure 4:**
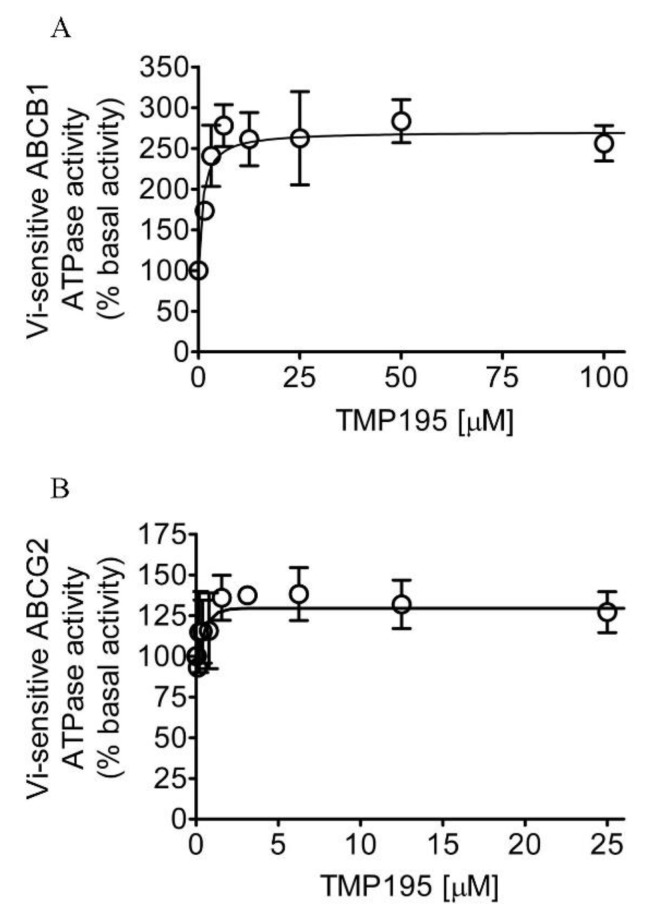
TMP195 stimulates ATPase activity of ABCB1 and ABCG2. The effect of TMP195 on vanadate-sensitive ATPase activity of (**A**) ABCB1 and (**B**) ABCG2 was determined by the endpoint P_i_ assay as described in the Section 4. Data are presented as a mean ± SD from at least three independent experiments as a percentage of basal activity taken as 100%.

Knowing that TMP195 modulates the drug transport function of ABCB1 and ABCG2, we further explored the interaction between TMP195 with the substrate-binding pockets of ABCB1 and ABCG2. Given that ABCB1- and ABCG2-mediated substrate transport is coupled to ATP hydrolysis [35,36], we examined the effect of TMP195 on vanadate (Vi)-sensitive ATPase activity of ABCB1 and ABCG2. We found that TMP195 stimulated the ATPase activity of ABCB1 and ABCG2 in a concentration-dependent manner. As shown in Figure 4A, TMP195 produced a three-fold maximal stimulation of ABCB1 ATPase activity and a half-maximal effective concentration (EC_50_) value of approximately 2 μM (basal, 89.2 ± 15.2 nmol P_i_/min/mg protein). On the other hand, TMP195 produced a 40% maximal stimulation of ABCG2 ATPase activity and an EC_50_ value of approximately 0.4 μM (basal, 174.5 ± 28.6 nmol P_i_/min/mg protein) (Figure 4B). These findings suggest that similar to other modulators, TMP195 also interacts at the drug-binding pocket of ABCB1 and ABCG2.

### 2.5. In Silico Docking Analyses Reveals that TMP195 Binds in the Drug-Binding Pocket of ABCB1 and ABCG2

**Figure 5:**
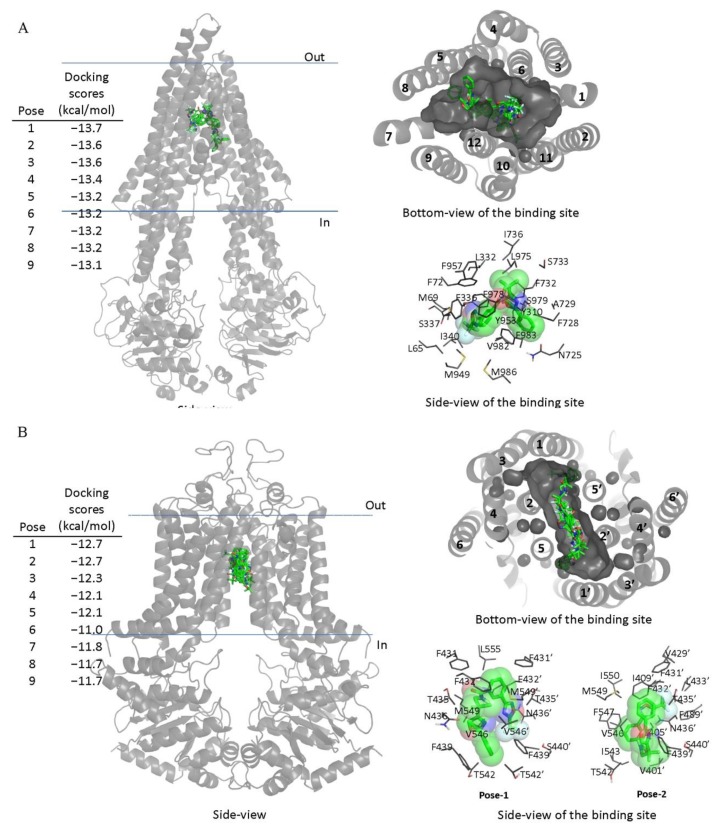
Docking of TMP195 in the drug-binding pockets of ABCB1 and ABCG2. Binding modes of TMP195 with (**A**) the inward-open structure of human ABCB1 (PDBID:6QEX) and (**B**) structure of ABCG2 (PDB: 5NJ3) obtained after exhaustive docking using AutoDock Vina software as described in Section 4. TMP195 is presented as a molecular model with atoms colored as carbon–green, nitrogen–blue, oxygen–red, florine–white. The docking scores of the first nine poses (tighter binding) are shown on the left. Cartoon representation shows all nine binding poses in the side- and bottom-view of each transporter. Binding-cavity is shown in dark gray from the bottom-view, and TM helix numbers are specified. TMP195 is presented in green sticks. The lowest energy poses for TMP195 in the transmembrane region of ABCB1 and ABCG2 are presented in dark gray lines to illustrate the residues that are within 4 Å of the ligand. Figures were prepared using the Pymol molecular graphics system, Version 1.7 Shrödinger, LLC.

Next, in order to elucidate the site of interaction between TMP195 and residues within the substrate-binding pockets of ABCB1 and ABCG2, we performed docking analysis of TMP195 with the inward-open structure of human ABCB1 (PDBID:6QEX) [37] and ABCG2 (PDBID:5NJ3) [38] as detailed in Section 4. Potential hydrophobic and aromatic interactions between TMP195 and the hydrophobic and aromatic residues located within the transmembrane domain (TMD) of ABCB1 (Figure 5A) and ABCG2 (Figure 5B) were identified via analysis of the lowest energy docking poses. These molecular modeling data suggest that TMP195 interacts directly with the substrate-binding pocket of ABCB1 and ABCG2.

### 2.6. The Overexpression of ABCB1 or ABCG2 Does not Affect the Chemosensitivity of Cancer Cells to TMP195

**Figure 6:**
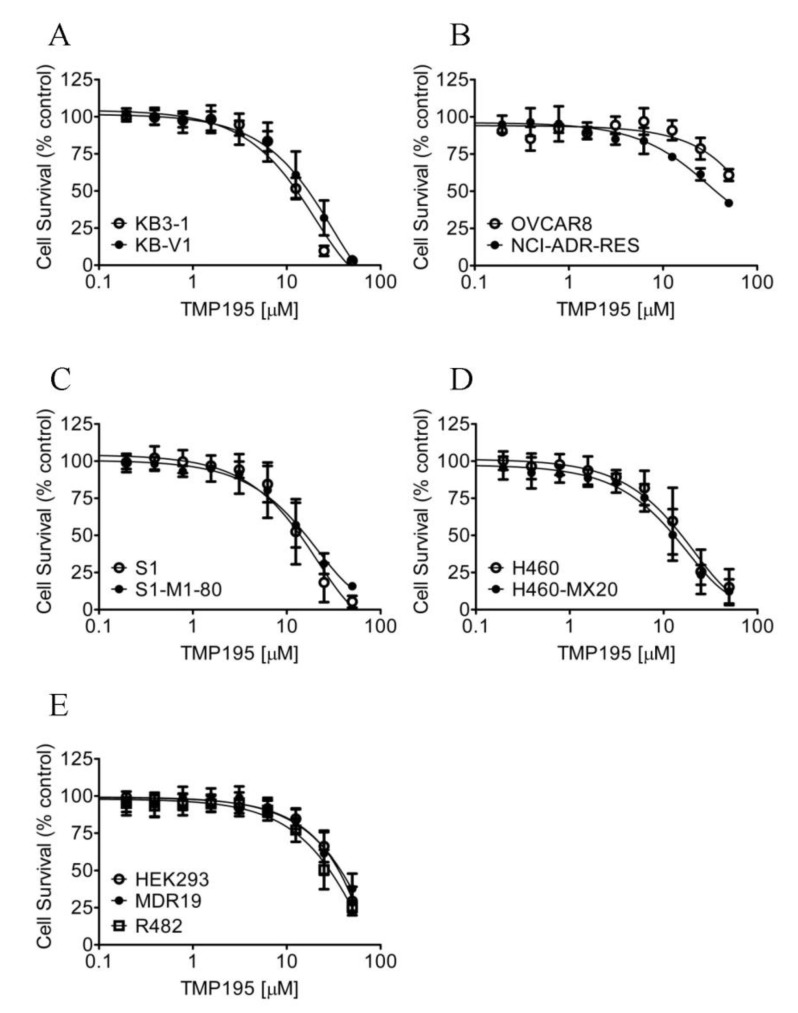
Drug-sensitive parental and multidrug-resistant cells overexpressing ABCB1 or ABCG2 are equally sensitive to TMP195. The cytotoxicity of TMP195 was determined in (**A**) drug-sensitive human epidermal cancer cell line KB-3-1 (open circles) and KB-V-1, the ABCB1-overexpressing multidrug-resistant variant (filled circles), (**B**) drug-sensitive human ovarian cancer cell line OVCAR-8 (open circles) and NCI-ADR-RES, the ABCB1-overexpressing multidrug-resistant variant (filled circles), (**C**) drug-sensitive human colon cancer cell line S1 (open circles) and S1-M1-80, the ABCG2-overexpressing multidrug-resistant variant (filled circles), (**D**) drug-sensitive human lung cancer cell line H460 (open circles) and H460-MX20, the ABCG2-overexpressing multidrug-resistant variant (filled circles), as well as (**E**) parental pcDNA-HEK293 cells (open circles) and HEK293 cells transfected with human ABCB1 (MDR19-HEK293, filled circles) or human ABCG2 (R482-HEK293, open squares). Points, mean values from at least three independent experiments; error bars; SD.

Considering that TMP195 interacts with the substrate-binding pockets of ABCB1 and ABCG2, and that the overexpression of these transporters is associated with reduced efficacy of several HDAC inhibitors in cancer cells [30,39,40], we thus compared the cytotoxicity of TMP195 in ABCB1- or ABCG2-overexpressing multidrug-resistant cell lines to their respective drug-sensitive parental cell lines (Figure 6). As summarized in Table 4, the resistance factor (RF) values, calculated by dividing the IC_50_ value of TMP195 in multidrug-resistant cell lines by the IC_50_ value of TMP195 in the respective drug-sensitive parental lines, are equal in all cell lines. Our results indicate that the overexpression of ABCB1 or ABCG2 is unlikely to reduce the susceptibility of cancer cells to TMP195.

## 3. Discussion

At present, the development of multidrug resistance during the course of cancer treatment associated with the upregulation and overexpression of ABC drug transporters in cancer cells remains an unsolved problem in modern chemotherapy [3,4,41]. The overexpression of ABCB1 and ABCG2 has been linked to the development of acquired drug resistance in numerous types of cancer, such as advanced non-small cell lung cancer [42], breast cancer [43], acute myelogenous leukemia (AML) and acute lymphocytic leukemia (ALL) [44,45,46], chronic myeloid leukemia (CML) [47], chronic lymphocytic leukemia (CLL) [48], and multiple myeloma (MM) [49,50,51,52,53,54,55]. Rather than developing synthetic inhibitors of ABCB1 and ABCG2, we and others have focused on the repositioning of molecularly targeted therapeutic agents to modulate the function and/or expression of ABCB1 and ABCG2, which is an alternative approach to resensitize multidrug-resistant cancer cells to conventional chemotherapeutic agents [11,17,20,34,56,57,58,59,60,61]. As a potent and highly selective class IIa HDAC inhibitor [21], TMP195 was shown able to trigger a therapeutic immune response that alters the tumor microenvironment, inhibits the proliferation and metastasis of breast tumors, and enhances the efficacy of chemotherapeutic agents and checkpoint blockade immunotherapy through modulation of macrophage phenotype [22]. More recently, TMP195 was described as a novel class of nuclear factor erythroid 2-related factor 2 (NRF2) activator that can be utilized to regulate cardiac redox homeostasis [62]. In the present study, we report an additional function of TMP195 in reversing ABCB1- and ABCG2-mediated multidrug resistance in cancer cells.

We first tested the chemosensitization effect of TMP195 in cells overexpressing ABCB1, ABCC1 or ABCG2. TMP195 at 5 μM was chosen as the highest tested concentration based on the cytotoxicity profile of TMP195 and a previous study reporting TMP195 not being cytotoxic in RT112 human bladder urothelial cancer cell line at this concentration [63]. We discovered that TMP195 resensitizes ABCB1-overexpressing cancer cells to paclitaxel, colchicine, and vincristine (Table 2), as well as resensitizes ABCG2-overexpressing cancer cells to mitoxantrone, SN-38, and topotecan (Table 3) in a concentration-dependent manner. Since we were unable to distinguish growth retardation from drug-induced cytotoxicity based on the 72 h cell proliferation assay alone, we thus examined the effect of TMP195 on drug-induced apoptosis in these cancer cells after a shorter treatment time of 48 h. We confirmed that TMP195 reversed ABCB1- and ABCG2-mediated drug resistance by enhancing drug-induced apoptosis in ABCB1- and ABCG2-overexpressing multidrug-resistant cancer cells (Figure 2). The inhibitory effect of TMP195 on ABCB1- and ABCG2-mediated drug transport, as well as its effect on the protein expression of ABCB1 and ABCG2 in multidrug-resistant cancer cells were examined to elucidate the potential mechanism(s) of chemosensitization by TMP195. We discovered that the drug transport function of ABCB1 and ABCG2 was significantly inhibited by TMP195. In contrast, the protein expression of ABCB1 and ABCG2 in multidrug-resistant cancer cells was unaffected by TMP195 for 72 h, indicating that the downregulation of ABCB1 or ABCG2 does not play a significant role in the chemosensitization of multidrug-resistant cancer cells by TMP195.

**Figure 7:**
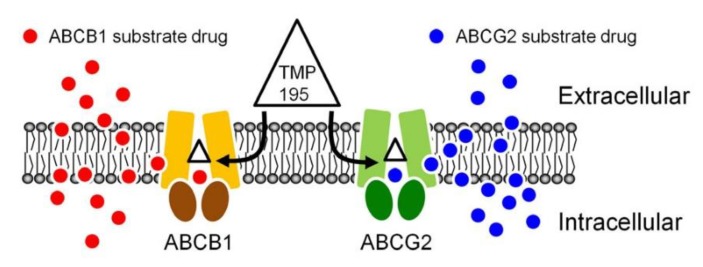
A simplified schematic diagram of TMP195 reversing drug resistance in cancer cells overexpressing ABCB1 or ABCG2 by attenuating the function of ABCB1 and ABCG2. The ATP-binding cassette proteins ABCB1 (brown) and ABCG2 (green) reduce intracellular drug concentration by actively transporting ABCB1 substrate drug (red circles) and ABCG2 substrate drug (blue circles) out of the cancer cell, which leads to multidrug resistant phenotype. By binding to the drug-binding pocket(s) of ABCB1 and ABCG2, TMP195 (triangle) attenuates the drug efflux function of both ABCB1 and ABCG2, thus restoring the chemosensitivity of cancer cells to these chemotherapeutic drugs.

The result of TMP195 stimulating the ATPase activity of ABCB1 and ABCG2 provided further insight into the binding interactions between TMP195 and the substrate-binding sites of ABCB1 and ABCG2. This as well as the in silico docking analysis using the inward-open conformation (the conformation of the transporter with binding of the substrate to the transmembrane region, of human ABCB1 and ABCG2) indicate that TMP195 binds to the substrate-binding pocket within the transmembrane regions of both ABCB1 and ABCG2. Therefore, although other possible mechanisms cannot be excluded, it is most likely that TMP195 reverses multidrug resistance mediated by ABCB1 and ABCG2 by competing directly with the binding of another substrate drug at the same site (Figure 7), which is consistent with the drug accumulation data. It is worth mentioning that several studies have reported that prolonged exposure of cancer cells to some HDAC inhibitors can lead to the induction of ABCB1 and/or ABCG2 and the MDR phenotype [64,65,66,67,68,69,70], and the overexpression of ABCB1 and/or ABCG2 has also been linked to reduced efficacy of numerous HDAC inhibitors [30,39,40,68,70,71,72]. For instance, studies have revealed that FK228 (romidepsin) is a substrate of both ABCB1 [68,71,72] and ABCG2 [70], and that continuous exposure to FK228 induces expression of ABCB1 [73] and ABCG2 [74] in cancer cells. In summary, although adverse drug–drug interaction may occur in combination therapy, our data suggest that combining conventional chemotherapeutic drugs with TMP195 may be a feasible therapeutic strategy to overcome cancer multidrug resistance associated with the overexpression of ABCB1 and/or ABCG2 and should be evaluated in future clinical studies.

## 4. Materials and Methods

### 4.1. Chemicals

RPMI-1640 medium, Dulbecco’s modified Eagle’s medium (DMEM), Iscove’s modified Dulbecco’s medium (IMDM), fetal calf serum (FCS), phosphate-buffered saline (PBS), trypsin-EDTA, penicillin, and streptomycin were purchased from Gibco, Thermo Fisher Scientific (Waltham, MA, USA). Tools Cell Counting (CCK-8) Kit was obtained from Biotools Co., Ltd. (Taipei, Taiwan). Annexin V-FITC Apoptosis Detection Kit was purchased from BD Pharmingen (San Diego, CA, USA). Verapamil, MK-571, Ko143, and all other chemicals were purchased from Sigma (St. Louis, MO, USA) unless stated otherwise. TMP195 was purchased from Selleckchem (Houston, TX, USA).

### 4.2. Cell Culture Conditions

The human embryonic kidney HEK293 cells, HEK293 cells stably transfected with human ABCB1 (MDR19-HEK293), and HEK293 cells stably transfected with wild-type human ABCG2 (R482-HEK293) were maintained in DMEM containing 2 mg/mL G418 [75]. Parental KB-3-1 human epidermal cancer cells were cultured in DMEM, and the ABCB1-overexpressing variant KB-V-1 cells were cultured in DMEM supplemented with 1 mg/mL vinblastine [76]. Parental OVCAR-8 human ovarian cancer cells and the ABCB1-overexpressing variant NCI-ADR-RES cells; parental H460 human non-small cell lung cancer cells and the ABCG2-overexpressing variant H460-MX20 cells; parental S1 human colon cancer cells and the ABCG2-overexpressing variant S1-M1-80 cells were maintained in RPMI-1640. H460-MX20 cells and S1-M1-80 cells were cultured in the presence of 20 nM mitoxantrone [77] or 80 µM mitoxantrone [78], respectively. Cell lines were maintained in medium supplemented with 10% FCS, 2 mM L-glutamine, and 100 units of penicillin/streptomycin/mL at 37 °C in 5% CO_2_ humidified air and maintained in drug-free medium for 7 days prior to assay.

### 4.3. Cell Viability Assay

The cytotoxicity of therapeutic drugs in respective drug-sensitive and drug-resistant cell lines was determined according to the method described by Ishiyama et al. [79]. Briefly, cells were seeded into each well of the 96-well flat-bottom plates and allowed to attach for 24 h, and each drug regimen was then added to each well and incubated at 37 °C in 5% CO_2_ humidified air for an additional 72 h. The cytotoxicity of drugs in HEK293 cells and transfectants was determined using CCK-8 reagent, whereas the cytotoxicity of drugs in attached cancer cells was determined using MTT reagent. The extent of reversal was determined by adding TMP195, verapamil, MK-571 or Ko143 to the cytotoxicity assays and presented as fold-reversal (FR) values, as described previously [26,75].

### 4.4. Apoptosis Assay

The concurrent annexin V–FITC and propidium iodide (PI) staining method was employed to quantify the percentage of apoptotic cell population induced by various drug regimens [80]. Briefly, cells were treated with DMSO, colchicine, topotecan, TMP195 or a combination of colchicine and TMP195 or topotecan and TMP195 for 48 h, then stained with 1.25 µg/mL annexin V–FITC and 0.1 mg/mL PI (BD PharMingen, Franklin Lanes, NJ, USA) and incubated for 15 min at room temperature. Labeled cells (10,000 cells per sample) were analyzed using FACScan equipped with CellQuest software (Becton-Dickinson Biosciences, San Jose, CA, USA) as described previously [20].

### 4.5. Fluorescent Drug Accumulation Assay

The intracellular accumulation of calcein-AM (a known substrate of ABCB1), calcein (a known substrate of ABCC1) or pheophorbide A (PhA) (a known substrate of ABCG2) was determined in respective cell lines in the presence or absence of 20 μM TMP195, 20 μM verapamil, 25 μM of MK-571 or 1 μM of Ko143 using a FACSort flow cytometer (Becton-Dickinson Biosciences, San Jose, CA, USA) as described previously [32,81]. Results were analyzed using CellQuest software and FlowJo software (Tree Star, Inc., Ashland, OR, USA) according to the method described by Gribar et al. [82].

### 4.6. Immunoblotting

The ABCB1- and ABCG2-overexpressing cancer cells were treated with DMSO (control) or increasing concentrations of TMP195 (1, 2, 3, and 5 μM) for 72 h before being harvested and subjected to SDS-polyacrylamide electrophoresis and Western blot immunoassay as described previously [75]. Blots were probed with primary antibody C219 (1:3000 dilution) for the detection of ABCB1, or BXP-21 (1:1000 dilution) for the detection of ABCG2, or α-tubulin (1:100,000 dilution) for the detection of the positive loading control tubulin. The horseradish peroxidase-conjugated goat anti-mouse IgG (1:100,000 dilution) was used as the secondary antibody. The enhanced chemiluminescence (ECL) kit (Merck Millipore, Billerica, MA, USA) was used for signal detection and visualization [75].

### 4.7. ATPase Assays

TMP195-stimulated vanadate (Vi)-sensitive ATPase activity of ABCB1 or ABCG2 was determined based on the endpoint P_i_ assay as described previously [83]. Briefly, membrane vesicles were purified by hypotonic lysis followed by differential centrifugation [84,85] from High-Five insect cells (Thermo Fisher Scientific, Waltman, MA, USA) infected with recombinant baculovirus containing ABCB1 or ABCG2 genes as previously described [86]. Membrane vesicles (0.1 µg total protein/µL, 50 µL) were incubated in the absence or presence of 0.3 mM sodium orthovanadate in ATPase buffer (50 mM MES-Tris pH 6.8, 50 mM KCl, 5 mM NaN_3_, 1 mM EGTA, 1 mM ouabain, 2 mM dithiothreitol). Basal ATPase activity was determined in the presence of 1% DMSO. TMP195 stocks were prepared in DMSO and added to the ATPase mix (1:100 dilution). After incubation of the samples for 3 min at 37 °C, 5 mM ATP was added. ATP hydrolysis was allowed to take place for 20 min after which the reaction was stopped by the addition of 50 µL of P_i_ reagent (1% ammonium molybdate in 2.5 N H_2_SO_4_ and 0.014% antimony potassium tartrate). The liberated inorganic phosphate was quantified by the addition of 150 µL of 0.33% sodium L-ascorbate. Absorbance at 880 nm was measured using a Spectramax^®^ (Molecular Devices, San Jose, CA, USA). The Vi-sensitive activity was calculated as the ATPase activity in the absence of vanadate minus the ATPase activity in the presence of vanadate, as described previously [83]. The ATPase activity in the presence of DMSO was defined as 100% basal activity. Experiments were repeated 3–5 times and the error bars presented as standard deviation.

### 4.8. Docking of TMP195 in the Drug-Binding Pocket of ABCB1 and ABCG2

The inward-open structure of human ABCB1 (PDBID:6QEX) [37] and the structure of ABCG2 (PDBID:5NJ3) [38] were used for docking of TMP195 with AutoDock Vina [87]. Transporters and ligands were prepared using MGLtools software package (Scripps Research Institute) [88,89]. The side chains of 36 residues, all located in the drug-binding pocket in the transmembrane region of ABCB1 were set as flexible. For docking in the drug-binding pocket of ABCB1, the following residues were set as flexible: L65, M68, M69, F72, Q195, W232, F303, I306, Y307, Y310, F314, F336, L339, I340, F343, Q347, N721, Q725, F728, F732, F759, F770, F938, F942, Q946, M949, Y953, F957, L975, F978, V982, F983, M986, Q990, F993, F994. The receptor grid was centered at x = 19, y = 53, and z = 3, and a box with inner box dimensions 40 Å × 40 Å × 44 Å was used to search for all the possible binding poses within the transmembrane region of the protein. In the case of ABCG2, the following residues were set as flexible: N393, A397, N398, V401, L405, I409, T413, N424, F431, F432, T435, N436, F439, S440, V442, S443, Y538, L539, T542, I543, V546, F547, M549, I550, L554, L555. The receptor grid was centered at x = 125, y = 125, and z = 130, and a box with inner box dimensions 34 Å × 30 Å × 50 Å was used. The exhaustiveness level was set at 100 for both proteins to ensure that the global minimum of the scoring function would be found considering the large box size and the number of flexible residues. Analysis of the docked poses was performed using Pymol molecular graphics system, Version 1.7 (Shrödinger, LLC, New York, NY, USA).

### 4.9. Quantification and Statistical Analysis

The experimental and IC_50_ values were calculated from at least three independent experiments and presented as mean ± standard deviation (SD) unless stated otherwise. GraphPad Prism (GraphPad Software, La Jolla, CA, USA) and KaleidaGraph (Synergy Software, Reading, PA, USA) software were used for curve plotting and statistical analysis. The difference between mean values of experimental and control or improvement in fit was analyzed by two-tailed Student’s *t*-test and labeled with asterisks as “statistically significant” if the probability, *p*, was less than 0.05.

## Figures and Tables

**Table 1:** Effect of TMP195 on drug resistance mediated by major ATP-binding cassette (ABC) drug efflux transporters in HEK293 cells transfected with ABCB1, ABCC1 or ABCG2.

Treatment	Concentration (μM)	Mean IC_50_^†^ ± SD and (FR ^‡^)	
		pcDNA-HEK293 (Parental) (nM)	MDR19-HEK293 (Resistant) (nM)
Paclitaxel	-	2.45 ± 0.47 (1.0)	1020.80 ± 176.45 (1.0)
+TMP195	1	2.01 ± 0.24 (1.2)	462.33 ± 84.71 ** (2.2)
+TMP195	2	1.87 ± 0.32 (1.3)	222.28 ± 26.22 ** (4.6)
+TMP195	3	2.19 ± 0.38 (1.1)	160.98 ± 35.26 ** (6.3)
+TMP195	5	1.91 ± 0.27 (1.3)	62.69 ± 6.33 *** (16.3)
+Verapamil	5	1.81 ± 0.41 (1.4)	10.03 ± 1.53 *** (101.8)
		pcDNA-HEK293 (parental) (nM)	R482-HEK293 (resistant) (nM)
Mitoxantrone	-	2.24 ± 0.38 (1.0)	65.08 ± 6.00 (1.0)
+TMP195	1	1.87 ± 0.34 (1.2)	20.53 ± 4.07 *** (3.2)
+TMP195	2	1.71 ± 0.36 (1.3)	11.86 ± 1.61 *** (5.5)
+TMP195	3	1.62 ± 0.24 (1.4)	12.42 ± 2.33 *** (5.3)
+TMP195	5	1.56 ± 0.29 (1.4)	6.70 ± 1.33 *** (9.7)
+Ko143	3	1.81 ± 0.32 (1.2)	3.60 ± 0.38 *** (18.1)
		pcDNA-HEK293 (parental) (nM)	MRP1-HEK293 (resistant) (μM)
Etoposide	-	175.37 ± 35.99 (1.0)	86.22 ± 11.07 (1.0)
+TMP195	1	185.10 ± 30.36 (0.9)	110.60 ± 18.86 (0.8)
+TMP195	2	236.17 ± 52.11 (0.7)	104.62 ± 16.93 (0.8)
+TMP195	3	258.68 ± 38.05 (0.7)	96.09 ± 29.32 (0.9)
+TMP195	5	172.29 ± 30.28 (1.0)	72.24 ± 9.20 (1.2)
+MK-571	25	160.23 ± 27.21 (1.1)	19.20 ± 1.47 *** (4.5)

Abbreviation: FR, fold-reversal. ^†^ IC_50_ values are mean ± SD calculated from dose-response curves obtained from at least three independent experiments using cytotoxicity assay as described in Section 4. ^‡^ FR values were calculated by dividing IC_50_ values of cells treated with a particular therapeutic drug in the absence of TMP195 or a respective reference inhibitor by IC_50_ values of cells treated with the same therapeutic drug in the presence of TMP195 or a respective reference inhibitor. * *p* < 0.05; ** *p* < 0.01; *** *p* < 0.001.

**Table 2:** Chemosensitizing effect of TMP195 on multidrug resistance mediated by ABCB1 in ABCB1-overexpressing human cancer cells.

Treatment	Concentration (μM)	Mean IC_50_^†^ ± SD and (FR ^‡^)	
		KB-3-1 (Parental) (nM)	KB-V-1 (Resistant) (nM)
Paclitaxel	-	2.21 ± 0.64 (1.0)	3204.25 ± 481.06 (1.0)
+TMP195	1	1.93 ± 0.41 (1.1)	1571.27 ± 217.65 ** (2.0)
+TMP195	2	1.90 ± 0.45 (1.2)	1082.46 ± 160.30 ** (3.0)
+TMP195	3	1.74 ± 0.34 (1.3)	656.68 ± 64.71 *** (4.9)
+TMP195	5	1.73 ± 0.39 (1.3)	306.75 ± 26.08 *** (10.4)
+Verapamil	5	1.83 ±0.43 (1.2)	65.68 ± 3.26 *** (48.8)
Colchicine		9.25 ± 4.23 (1.0)	2251.10 ± 372.67 (1.0)
+TMP195	1	9.46 ± 4.45 (1.0)	1021.63 ± 103.07 ** (2.2)
+TMP195	2	8.35 ± 3.31 (1.1)	588.27 ± 87.86 ** (3.8)
+TMP195	3	7.35 ± 3.18 (1.3)	326.59 ± 70.84 *** (6.9)
+TMP195	5	7.86 ± 3.82 (1.2)	206.80 ± 32.01 *** (10.9)
+Verapamil	5	6.61 ± 3.20 (1.4)	204.97 ± 35.01 *** (11.0)
Vincristine	-	0.53 ± 0.11 (1.0)	897.11 ± 108.89 (1.0)
+TMP195	1	0.58 ± 0.15 (0.7)	450.57 ± 39.31 ** (2.0)
+TMP195	2	0.54 ± 0.13 (0.9)	318.47 ± 35.40 *** (2.8)
+TMP195	3	0.52 ± 0.13 (1.0)	185.27 ± 10.62 *** (4.8)
+TMP195	5	0.45 ± 0.13 (1.2)	67.85 ± 3.91 *** (13.2)
+Verapamil	5	0.19 ± 0.05 ** (2.8)	8.23 ± 1.13 *** (109.0)
Treatment	Concentration (μM)	OVCAR-8 (parental) (nM)	NCI-ADR-RES (resistant) (μM)
Paclitaxel	-	5.42 ± 1.07 (1.0)	8.52 ± 1.83 (1.0)
+TMP195	1	5.11 ± 0.92 (1.1)	6.24 ± 1.00 (1.4)
+TMP195	2	4.93 ± 1.02 (1.1)	3.18 ± 0.58 ** (2.7)
+TMP195	3	4.50 ± 0.78 (1.2)	1.88 ± 0.22 ** (4.5)
+TMP195	5	4.36 ± 0.82 (1.2)	0.92 ± 0.21 ** (9.3)
+Verapamil	5	3.75 ± 0.85 (1.4)	0.34 ± 0.04 ** (25.1)
Colchicine	-	19.66 ± 6.36 (1.0)	2.98 ± 0.60 (1.0)
+TMP195	1	21.53 ± 7.24 (0.9)	2.97 ± 0.81 (1.0)
+TMP195	2	21.11 ± 6.95 (0.9)	2.16 ± 0.49 (1.4)
+TMP195	3	20.58 ± 6.90 (1.0)	1.63 ± 0.37 * (1.8)
+TMP195	5	19.67 ± 6.36 (1.0)	1.02 ± 0.31 ** (2.9)
+Verapamil	5	15.76 ± 5.91 (1.2)	0.63 ± 0.15 ** (4.7)
Vincristine	-	2.84 ± 0.43 (1.0)	4.64 ± 0.99 (1.0)
+TMP195	1	2.78 ± 0.32 (1.0)	3.18 ± 0.65 (1.5)
+TMP195	2	2.46 ± 0.33 (1.2)	1.95 ± 0.39 * (2.4)
+TMP195	3	2.29 ± 0.37 (1.2)	1.48 ± 0.40 ** (3.1)
+TMP195	5	2.28 ± 0.28 (1.2)	0.77 ± 0.25 ** (6.0)
+Verapamil	5	0.85 ± 0.10 ** (3.3)	0.15 ± 0.03 ** (30.1)

Abbreviation: FR, fold-reversal. ^†^ IC_50_ values are mean ± SD calculated from dose-response curves obtained from at least three independent experiments using cytotoxicity assay as described in Section 4. ^‡^ FR values were calculated by dividing IC_50_ values of cells treated with a particular therapeutic drug in the absence of TMP195 or verapamil by IC_50_ values of cells treated with the same therapeutic drug in the presence of TMP195 or verapamil. * *p* < 0.05; ** *p* < 0.01; *** *p* < 0.001.

**Table 3:** Chemosensitizing effect of TMP195 on multidrug resistance mediated by ABCG2 in ABCG2-overexpressing human cancer cells.

Treatment	Concentration (μM)	S1 (Parental) (nM)	S1-M1-80 (Resistant) (μM)
Mitoxantrone	-	12.76 ± 3.59 (1.0)	83.89 ± 8.40 (1.0)
+TMP195	1	10.64 ± 1.81 (1.2)	29.44 ± 7.48 ** (2.8)
+TMP195	2	11.11 ± 1.65 (1.1)	14.60 ± 4.86 *** (5.7)
+TMP195	3	9.40 ± 1.63 (1.4)	5.64 ± 2.09 *** (14.9)
+TMP195	5	8.15 ± 1.44 (1.6)	3.10 ± 1.04 *** (27.1)
+Ko143	3	12.14 ± 2.97 (1.1)	0.84 ± 0.13 *** (99.9)
SN-38	-	2.45 ± 0.35 (1.0)	5.14 ± 1.19 (1.0)
+TMP195	1	2.02 ± 0.29 (1.2)	1.32 ± 0.28 ** (3.9)
+TMP195	2	1.96 ± 0.27 (1.3)	0.63 ± 0.10 ** (8.2)
+TMP195	3	1.90 ± 0.23 (1.3)	0.43 ± 0.08 ** (11.8)
+TMP195	5	1.83 ± 0.23 (1.3)	0.27 ± 0.05 ** (18.9)
+Ko143	3	2.14 ± 0.30 (1.1)	0.06 ± 0.01 ** (85.7)
Topotecan	-	117.54 ± 40.80 (1.0)	9.90 ± 2.35 (1.0)
+TMP195	1	100.80 ± 38.21 (1.2)	3.17 ± 0.79 ** (3.1)
+TMP195	2	71.82 ± 28.62 (1.6)	2.01 ± 0.49 ** (4.9)
+TMP195	3	71.51 ± 27.76 (1.6)	1.13 ± 0.31 ** (8.8)
+TMP195	5	58.92 ±24.87 (2.0)	0.98 ± 0.28 ** (10.1)
+Ko143	3	86.75 ± 33.89 (1.4)	0.39 ± 0.06 ** (25.4)
Treatment	Concentration (μM)	H460 (parental) (nM)	H460-MX20 (resistant) (nM)
Mitoxantrone	-	12.75 ± 1.38 (1.0)	1385.40 ± 109.51 (1.0)
+TMP195	1	14.85 ± 4.35 (0.9)	474.17 ± 62.44 *** (2.9)
+TMP195	2	9.37 ± 2.18 (1.4)	477.63 ± 129.56 *** (2.9)
+TMP195	3	16.00 ± 3.72 (0.8)	417.30 ± 83.81 *** (3.3)
+TMP195	5	12.31 ± 2.79 (1.0)	332.69 ± 87.33 *** (4.2)
+Ko143	3	15.31 ± 3.38 (0.8)	168.99 ± 50.81 *** (8.2)
SN-38	-	6.60 ± 1.22 (1.0)	598.64 ± 198.78 (1.0)
+TMP195	1	5.80 ± 1.20 (1.1)	108.83 ± 37.21 * (5.5)
+TMP195	2	4.45 ± 0.93 (1.5)	76.26 ± 29.92 * (7.8)
+TMP195	3	5.33 ± 1.44 (1.2)	80.39 ± 25.85 * (7.4)
+TMP195	5	5.09 ± 1.61 (1.3)	67.97 ± 24.63 * (8.8)
+Ko143	3	4.36 ± 1.05 (1.5)	5.23 ± 1.48 ** (114.46)
Topotecan	-	39.40 ± 6.12 (1.0)	1329.94 ± 238.00 (1.0)
+TMP195	1	26.95 ± 5.01 (1.5)	685.75 ± 187.43 * (1.9)
+TMP195	2	21.26 ± 4.06 * (1.9)	386.7 ± 124.95 ** (3.4)
+TMP195	3	23.92 ± 4.39 * (1.6)	270.72 ± 76.57 ** (4.9)
+TMP195	5	23.35 ± 5.17 * (1.7)	164.61 ± 55.23 ** (8.1)
+Ko143	3	17.98 ± 3.21 ** (2.2)	99.41 ± 28.67 *** (13.4)

Abbreviation: FR, fold-reversal. ^†^ IC_50_ values are mean ± SD calculated from dose-response curves obtained from at least three independent experiments using cytotoxicity assay as described in Section 4. ^‡^ FR values were calculated by dividing IC_50_ values of cells treated with a particular therapeutic drug in the absence of TMP195 or Ko143 by IC_50_ values of cells treated with the same therapeutic drug in the presence of TMP195 or Ko143. * *p* < 0.05; ** *p* < 0.01; *** *p* < 0.001.

**Table 4:** Cytotoxicity of TMP195 in human cell lines overexpressing ABCB1 or ABCG2.

Cell Line	Type	Transporter Expressed	IC_50_ (μM) ^†^	RF ^‡^
KB-3-1	epidermal	-	10.53 ± 3.23	1.0
KB-V1	epidermal	ABCB1	13.74 ± 2.91	1.3
OVCAR-8	ovarian	-	>35	1.0
NCI-ADR-RES	ovarian	ABCB1	>35	1.0
S1	colon	-	12.18 ± 2.90	1.0
S1-M1-80	colon	ABCG2	11.98 ± 1.22	1.0
H460	lung	-	14.09 ± 2.45	1.0
H460-MX20	lung	ABCG2	12.17 ± 1.95	0.9
pcDNA-HEK293	-	-	29.52 ± 7.63	1.0
MDR19-HEK293	-	ABCB1	30.98 ± 7.76	1.0
R482-HEK293	-	ABCG2	28.64 ± 5.07	1.0

Abbreviation: RF, resistance factor. ^†^ IC_50_ values are mean ± SD calculated from dose-response curves obtained from at least three independent experiments using cytotoxicity assay as described in Section 4. RF ^‡^ values were obtained by dividing the IC_50_ value of TMP195 in ABCB1- or ABCG2-overexpressing multidrug-resistant cell lines by the IC_50_ value of TMP195 in respective drug-sensitive parental cell lines. * *p* < 0.05; ** *p* < 0.01; *** *p* < 0.001.

## Abbreviations

ABC	ATP-binding cassette
ALL	Acute lymphocytic leukemia
AML	Acute myelogenous leukemia
BCRP	Breast cancer resistance protein
CCK-8	Cell Counting Kit-8
CLL	Chronic lymphocytic leukemia
CML	Chronic myeloid leukemia
ECL	Enhanced chemiluminescence
FR	Fold-reversal
HDAC	Histone deacetylase
MDR	Multidrug resistance
MM	Multiple myeloma
P-gp	P-glycoprotein
RF	Resistance factor
SD	Standard deviation
Vi	Sodium orthovanadate
